# Prognostic Impact of Adenosine Receptor 2 (A2aR) and Programmed Cell Death Ligand 1 (PD-L1) Expression in Colorectal Cancer

**DOI:** 10.1155/2019/8014627

**Published:** 2019-06-03

**Authors:** Zhaoying Wu, Lin Yang, Linsen Shi, Hu Song, Peicong Shi, Ting Yang, Ruizhi Fan, Tao Jiang, Jun Song

**Affiliations:** ^1^Xuzhou Medical University, Xuzhou 221002, Jiangsu Province, China; ^2^Department of General Surgery, The Affiliated Hospital of Xuzhou Medical University, Xuzhou 221002, Jiangsu Province, China; ^3^Institute of Digestive Diseases of Xuzhou Medical University, Xuzhou 221002, Jiangsu Province, China

## Abstract

**Background:**

Programmed cell death ligand 1 (PD-L1) is a key inhibitor to the immune response by binding to the specific receptor PD-1. Adenosine receptor 2 (A2aR) can play an immunosuppressive role in tumor microenvironment by binding to its ligand adenosine (ADO). However, the expression of these two markers has been rarely studied in colorectal cancer simultaneously.

**Materials and Methods:**

We, respectively, collected tumor and adjacent nontumor tissue specimens of 204 patients with colorectal cancer. The expressions of PD-L1 and A2aR were detected by immunohistochemistry. The association among their expressions with clinicopathological characteristics and prognostic parameters were analyzed as well.

**Results:**

The expressions of PD-L1 and A2aR in tumor tissues were both higher than those in matched adjacent nontumor tissues. PD-L1 expression was significantly correlated with lymph node metastasis and tumor TNM stage. A2aR expression was significantly correlated with tumor size, depth of tumor invasion, and TNM stage. Univariate analysis showed that the high expressions of PD-L1 and A2aR were inversely correlated with the overall survival, respectively. Multivariate analysis further confirmed that both of them were independent prognostic markers for patients.

**Conclusion:**

The results of this study suggested that the high expressions of PD-L1 and A2aR were associated with a poor prognosis of colorectal cancer. Coinhibition of these two proteins may be a new breakthrough in the treatment of this disease.

## 1. Introduction

Colorectal cancer is one of the most common malignant tumors in the world. It is the third most common cause of cancer-related death in women and the fourth most frequent cause of that in men [1]. Radical surgery combined with chemoradiotherapy is currently the main treatment method for this disease [2]. Although almost 90% people with early-stage tumors could have a 5-year life expectancy after standardized treatment, still up to 40% of the patients would experience local recurrence or distant metastasis [3], which prompts oncologists to explore further potentially effective treatments, such as immunotherapy.

Tumor immunotherapy was listed as one of the top ten scientific breakthroughs by Science magazine in 2013 [4]. The purpose of tumor immunotherapy is to stimulate or mobilize the immune system of the body and enhance the antitumor immunity of tumor microenvironment, so as to inhibit and kill tumor cells [5].

Programmed death ligand 1 (PD-L1) is a costimulatory molecule expressed by antigen-presenting cells and is thought to have the function to inhibit the T lymphocyte molecule. It belongs to the B7 superfamily and is a type I transmembrane protein which contains a transmembrane region and two extracellular domains, IgC and IgV [6, 7]. PD-L1 can downregulate the proliferation of antigen-stimulated lymphocytes and decrease the production of cytokines by binding to the specific receptor PD-1, which ultimately leads to the emergence of immune tolerance [8, 9]. Upregulated-expression of PD-L1 has been found in many types of tumors, including non-small-cell lung cancer, malignant melanoma, breast cancer, and ovarian cancer and it is thought that the expression of PD-L1 is related to the prognosis of these diseases [10–13]. Shi et al. found that the positive expression of PD-L1 protein in colorectal cancer patients was significantly associated with increased tumor-related mortality, suggesting that high PD-L1 expression could be used as a biomarker for poor prognosis [14]. However, Droeser et al. believed that the strong expression of PD-L1 in colorectal cancer was associated with early T stage, low tumor grade, absence of lymph node metastases and vascular invasion, and improved survival of patients [15]. Therefore, it is meaningful to further clarify the correlation between the expression status of PD-L1 and the prognosis of patients.

A2a adenosine receptor (A2aR) is one of the four receptors for adenosine (ADO) with the other three including A1R, A2bR, and A3R, and all of them belong to the superfamily of G-protein coupled receptors [16]. ADO, a metabolite of ATP, is involved in physiological processes such as energy metabolism and nucleic acid synthesis intracellularly and is an important signal transduction factor intercellularly [17]. However, due to insufficient lysis of ATP in the hypoxic microenvironment of tumor tissue, the hypoxic-inducible factor *α* induces the excessive expression of extracellular nucleotidase (CD39; CD73), which can decompose ATP into ADO, resulting in significant increase of ADO [18]. In the immune microenvironment, ADO is combined with A2aR to activate intracellular adenylate cyclase so that cAMP, the second messenger, is produced. Then cAMP can inhibit the immunological effect of human body through the cAMP, protein kinase A (PKA), and lymphocyte specific tyrosine kinase (Lck)/sarcoma gene protein kinase (Src kinase) pathway. The whole process can not only inhibit the antitumor immune response of immune effector cells, but also improve the polarization and proliferation of immunosuppressive cells, thus finally enabling the tumor to get immune escape [19, 20]. In 2006, Ohta et al. demonstrated the important role of A2aR in regulating the immune response of adenosine-regulated effector T cell for the first time. It was demonstrated that the congenital defected of A2aR in mice with melanoma could enhance the function of CD8^+^ T cells, decrease tumor growth and angiogenesis and inhibit tumor metastasis [21]. Recently, Waickman et al. have showed that deletion of the A2aR could promote antitumor responses of immune cells such as CD4^+^ T cells and CD8^+^ T cells by blocking ADO-A2aR-cAMP intracellular signaling [22].

So far, the correlation between PD-L1 expression and prognosis of colorectal cancer remains controversial, and the expression status of A2aR and its clinical significance in colorectal cancer has not even been investigated. Therefore, the aim of this study was to evaluate the expressions of PD-L1 and A2aR proteins in human colorectal tumor tissues and adjacent nontumor tissues by using immunohistochemistry (IHC), and to further investigate whether PD-L1 and A2aR expressions are associated with the clinicopathological features and prognosis of patients, thereby finding potential prognostic indicators and treatment strategies for this disease.

## 2. Material and Methods

### 2.1. Patients and Specimens

Objects of the study consisted of 204 colorectal cancer patients who were consecutively enrolled in the Affiliated Hospital of Xuzhou Medical University (Xuzhou, People's Republic of China) from June 2015 to June 2018. All patients have accepted surgical treatment in the hospital and have been diagnosed with colorectal carcinoma according to postoperative pathology. No patient has received chemotherapy or radiotherapy before surgery. All patients have gone through the standard treatments such as complete mesocolic excision (CME) and total mesorectal excision (TME); all tumors diagnosed as stage II and above according to postoperative pathology were treated with first-line chemotherapy for 6 cycles on the basis of NCCN guideline. Only 7 patients did not complete standard chemotherapy due to death. Experimental specimens were obtained by an experienced histopathologist in surgical operation. Matched nontumor tissue specimens were taken more than 5 cm away from the tumor margin. Clinicopathological features collected for each patient included gender, tumor size, tumor differentiation, tumor stage, etc. At recruitment, a consent form was signed by each subject. The study was approved by the ethics committee of the Affiliated Hospital of Xuzhou Medical University. Patients who were recruited in the study have been followed-up for 6 to 36 months after surgery, and the details of any postoperative recurrence or mortality were recorded accordingly.

### 2.2. Immunohistochemistry

The specimen was embedded in paraffin wax and cut into 3 mm-thick sections by a microtome. The tissue sections were first dewaxed by xylene three times and then dehydrated with graded ethanol. For antigen retrieval, the samples treated with PD-L1 antibody were heated for 20 min in an ethylenediaminetetraacetic acid (EDTA) buffer (pH = 8; Abcam, Cambridge, UK), and the sections treated with A2aR antibody were heated for 20 min in a citrate buffer (pH = 6). Subsequently, the sections were treated with 3% hydrogen peroxide for 15 minutes. All sections were incubated with anti-human PD-L1 monoclonal antibody (1:150; Abcam, Cambridge, UK) and anti-human A2aR monoclonal antibody (1:150; Abcam, Cambridge, UK) overnight at 4°C and then labeled with the HRP second antibody (SP-9001, Beijing Sequoia Jinqiao, Beijing, China) at room temperature for 1h. Reactivity was detected by using DAB reagent sets (Beijing Sequoia Jinqiao, Beijing, China), and the cells were counterstained with hematoxylin. As a negative control, the primary antibody was replaced by phosphate-buffered saline (PBS). Both PD-L1 and A2aR are mainly localized in the cell membrane. The positive group of PD-L1 was defined as greater than 1% of stained cells [23, 24]. The remaining cases were defined as the negative group. A semiquantitative integral method was applied to evaluate the expression of A2aR. The staining intensity of A2aR protein expression was scored as 0 (no staining); 1 (weak staining); 2 (moderate staining); and 3 (strong staining). The percentage area of positively stained cells within the visual field was classified as 0 (<5%); 1 (5–25%); 2 (26–50%); 3 (51–75%); or 4 (>75%). The evaluation was expressed as a product of the score of positive rate and staining intensity, defining a score of ≥6 as positive expression. Nikon DR-Si2 cell count software and digital image analysis were used for the evaluation of stain described above, and the results were verified by two senior pathologists who were blind to the clinicopathological data.

### 2.3. Statistical Analysis

SPSS 22.0 statistical package software was used for statistical analysis. The relationship between PD-L1 and A2aR expression in tumor issues and clinicopathological characteristics of patients was analyzed by *χ*^2^ test. The correlation between A2aR and PD-L1 expression was evaluated by Spearman correlation test. For prognostic factors, the univariate analysis was conducted by Kaplan-Meier method and log-rank test and multivariate analysis were performed by Cox proportional hazard model. P <0.05 was defined as the statistically significant.

## 3. Results

### 3.1. Clinicopathological Findings

204 patients made up of 124 males and 80 females were enrolled in the study with a mean age of 65.5 (25-89). Among the patients, 132 cases of tumor were located in colon, and the rest 72 cases of tumor were located in rectum. There were 104 cases with tumor size less than or equal to 5 cm and 100 cases with tumor size greater than 5 cm. This study included 30 TNM stage I cases, 76 TNM stage II cases, 79 TNM stage III cases, and 19 TNM stage IV cases. There were 18 cases of well-differentiated tumors, 164 cases of moderately differentiated tumors, and 22 cases of poorly differentiated tumors. 45 cases of the total 204 patients died in the course of follow-up due to deterioration of the disease. The median overall survival (OS) time was 22 (2-36) months. The detailed clinicopathological characteristics of the patients are listed in Table 2.

### 3.2. Expression Status of PD-L1 and Its Association with Clinicopathological Characteristics

**Figure 1:**
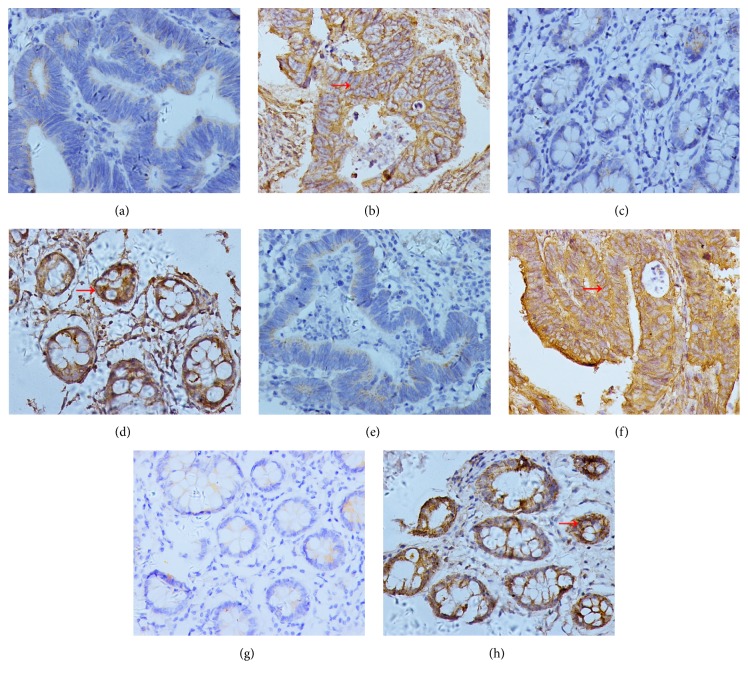
Assessment of protein expressions of PD-L1 and A2aR by IHC staining (400x original magnification). (a) The negative expression of PD-L1 in colorectal cancer tissues. (b) The positive expression of PD-L1 in colorectal cancer tissues (the red arrow indicates the tumor cell with positive expression of PD-L1). (c) The negative expression of PD-L1 in matched adjacent nontumor tissues. (d) The positive expression of PD-L1 in matched adjacent nontumor tissues (the red arrow indicates the cell in adjacent nontumor tissues with positive expression of PD-L1). (e) The negative expression of A2aR in colorectal cancer tissues. (f) The positive expression of A2aR in colorectal cancer tissues (the red arrow indicates the tumor cell with positive expression of A2aR). (g) The negative expression of A2aR in matched adjacent nontumor tissues. (h) The positive expression of A2aR in matched adjacent nontumor tissues (the red arrow indicates the cell in adjacent nontumor tissues with positive expression of A2aR). Notes: PD-L1 is mainly expressed on the cytomembrane. The positive expression of PD-L1 was detected in 84 (41.2%) of 204 tumor specimens and in 46 (22.5%) of the 204 matched adjacent nontumor tissues. A2aR is also mainly expressed on the cytomembrane. The positive expression of A2aR was detected in 108 (52.9%) of 204 tumor specimens and in 78 (38.2%) of the 204 matched adjacent nontumor tissues.

The representative expression status of PD-L1 in tumor tissues and adjacent nontumor tissues were displayed in Figures 1(a), 1(b), 1(c), and 1(d), respectively. PD-L1 was mainly expressed on the cytomembrane as shown in the picture. The positive expression of PD-L1 was detected in 84 (41.2%) of 204 tumor specimens and in 46 (22.5%) of the 204 matched adjacent nontumor tissues. The expression status of PD-L1 in tumor was significantly different from that in adjacent nontumor tissues (p<0.001, Table 1). The association between PD-L1 expression in tumor specimens and clinicopathological features of colorectal cancer patients was presented in Table 2. PD-L1 expression was significantly correlated with lymph node metastasis (p=0.006) and tumor TNM stage (p=0.014). There was no significant association between PD-L1 positive status in tumor tissues and other clinicopathological features of patients.

### 3.3. Expression Level of A2aR and Its Association with Clinicopathological Features

Representative immunohistochemical A2aR staining patterns in tumor tissues and adjacent nontumor tissues were shown in Figures 1(e), 1(f), 1(g), and 1(h), respectively. A2aR was also mainly expressed on the cytomembrane. The expression of A2aR was detected in 108 (52.9%) of 204 tumor specimens and in 78 (38.2%) of the 204 matched adjacent nontumor tissues. The expression of A2aR in tumor tissues was significantly different from that in adjacent nontumor tissues (p=0.003, Table 1). The association between A2aR expression in tumor and clinicopathological features of patients was presented in Table 2. The expression of A2aR was significantly correlated with tumor size (p=0.024), depth of tumor invasion (p=0.011) and TNM stage (p=0.005).

### 3.4. Correlation Analysis of the Expression of PD-L1 and A2aR in Colorectal Cancer Tissues

**Figure 2:**
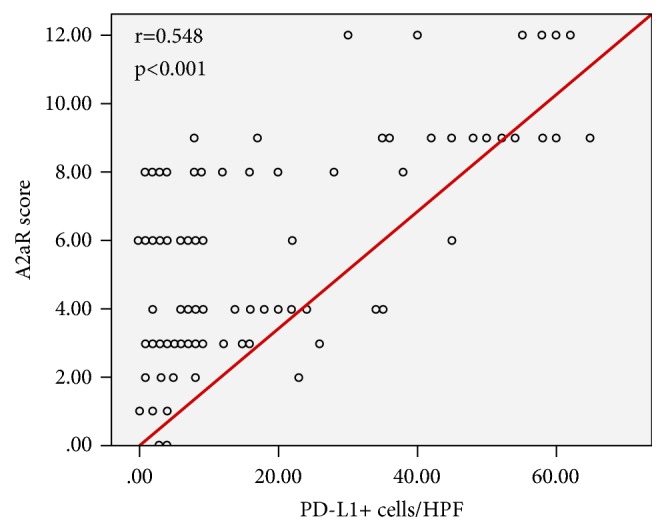
Relationship between the expressions of PD-L1 and A2aR in colorectal cancer tissues. Notes: the expression of PD-L1 in colorectal cancer was positively correlated with the expression of A2aR (r=0.548, p<0.001).

PD-L1 expression was positive in 84 cases, of which 56 cases were also positive for A2aR (66.7%), and the remaining 28 cases were negative for A2aR (33.3%). Of the 120 PD-L1-negative expression specimens, 68 cases were also negative for A2aR (56.7%), while the remaining 52 cases were positive for A2aR (43.3%). The expression of PD-L1 was positively correlated with the expression of A2aR in tumor (r=0.548, p<0.001) (Figure 2; Table 3).

### 3.5. Univariate and Multivariate Analysis of Prognostic Parameters in Colorectal Cancer Patients

**Figure 3:**
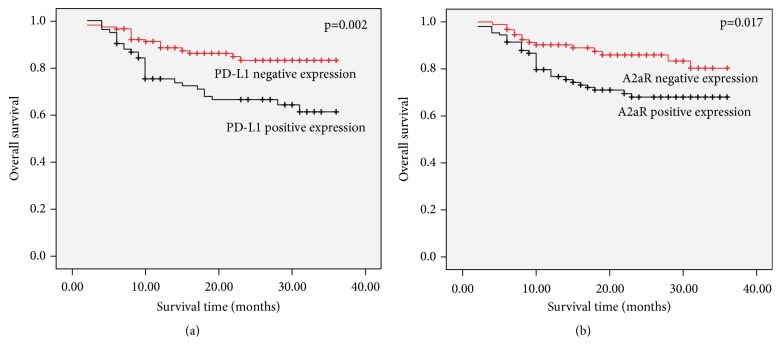
Different survival time of PD-L1-positive status and A2aR-positive status in colorectal cancer. Notes: PD-L1-positive status (P=0.002) (Figure 3(a)) and A2aR -positive status (P=0.017) (Figure 3(b)) were significantly correlated to OS (P < 0.05).

We further analyzed the correlations between clinicopathological features and overall survival (OS) by using the Kaplan-Meier method (Table 4). Depth of tumor invasion (p=0.025), lymph node metastasis (p<0.001), distant metastasis (p<0.001), tumor TNM stage (p<0.001), PD-L1-positive status in tumor (p=0.002, Figure 3(a)), and A2aR-positive status in tumor (p=0.017, Figure 3(b)) were significantly correlated to OS (p<0.05). However, there was no correlation between OS and patients' age (p=0.190) or sex (p=0.614), tumor location (p=0.845), tumor size (p=0.158), and tumor differentiation (p=0.661). Next, we selected potential prognostic factors based on univariate results (p<0.05) to conduct multivariate analysis by using Cox proportional hazards regression models (Table 5). Positive PD-L1 status (HR=1.914, 95% CI:1.031-3.553, p=0.040), positive A2aR expression (HR=2.400, 95% CI: 1.264-4.558, p=0.007), distant metastasis (HR=15.151, 95% CI: 6.502-35.303, p<0.001), and TNM stage (HR=4.195, 95% CI: 1.869-9.415, p=0.001) could be independent prognostic predictors for colorectal cancer patients. However, depth of tumor invasion (p=0.243) and lymph node metastasis (p=0.358) were not independent predictors for prognosis of disease.

## 4. Discussion

In recent years, with the great success of immunotherapy in malignant melanoma, lung cancer, and other tumors [25, 26], anticancer treatment has gradually entered a new stage of immunotherapy. Exploring pathogenic factors and even immunotherapy targets related to the progression of colorectal cancer can usher in new breakthroughs in the diagnosis and treatment of this disease.

PD-L1, also known as B7-H1 or CD274, is the ligand of PD-1. PD-1/PD-L1 signaling pathway can not only affect the antigen-presenting ability of dendritic cells, but also inhibit the immune function of T lymphocytes by limiting the activation of T cells and reducing the production of cytokines [27, 28]. In addition to expression in lymphocytes, macrophages, and the like, high expression of PD-L1 can also be detected in various cancer cells such as malignant melanoma, lung cancer, gastric cancer, breast cancer, hepatocellular carcinoma, bladder cancer, pancreatic cancer, and glioma [29]. The binding of PD-L1 on tumor cells to PD-1 on lymphocytes can lead to immune escape of tumor cells and ultimately promote the generation and development of tumors by inhibiting the release of cytokines, restricting lymphocyte function, and inducing lymphocyte apoptosis [30]. Some clinical studies have shown that the expression of PD-L1 is closely related to tumor size, lymph node metastasis, tumor stage, and overall survival [31]. Recently, Zhou et al. showed that PD-L1 is highly expressed in breast invasive ductal carcinoma, and the expression of PD-L1 in tumor cells is an independent prognostic factor for progression-free survival of this disease [32]. Wang et al. retrospectively analyzed 146 patients with esophageal squamous cell carcinoma (ESCC) to investigate the relationship between the expression of PD-L1 and infiltration of CD8^+^ T cells in tumor immune microenvironment with the prognosis of ESCC patients, and it was found that PD-L1 expression was significantly associated with poor overall survival; however, CD8^+^ T cell infiltration is not a risk factor for prognosis [33]. In the present study, we found that PD-L1 expression has a higher level in colorectal cancer tissues compared with that in adjacent nontumor tissues (p<0.001), and the positive status of PD-L1 is closely related to the lymph node metastasis (p=0.006) and tumor TNM stage (p=0.014). Moreover, univariate survival analysis has showed that positive expression of PD-L1 was inversely correlated with OS (p=0.002) and multivariate survival analysis has further confirmed that positive status of PD-L1 is an independent factor for poor prognosis of patients (HR=1.914, 95% CI:1.031-3.553, p=0.040), indicating that PD-L1 might play an important role in the generation and development of colorectal cancer.

Immune escape is now a widely recognized mechanism for tumor development. ADO-A2aR signaling is a well-established immunosuppressive pathway which is conducive to tumor immune evasion. Previous studies have indicated that A2aR is overexpressed in various tumor cells such as human breast cancer MCF-7 cells [34], non-small-cell lung cancer cells [35], U87MG human glioblastoma cells [36], and A375 melanoma cells [37]. Etique et al. have found that A2aR was overexpressed in the hormone-dependent breast cancer cell line MCF-7 by RT-PCR analyses and immunocytochemistry experiments, and further research confirmed that activation of A2aR by the selective agonist CGS21680 was able to stimulate proliferation of MCF-7 breast cancer cells [34]. In another study, the positive expression of A2aR was shown in non-small-cell lung cancer cells by immunohistochemistry, and the application of A2aR antagonists not only could downregulate the growth of tumor-associated fibroblasts and cancer cell in vitro, but also inhibited the growth of human tumor xenografts in mice [35]. Beavis et al. revealed that A2aR antagonist could effectively reduce the metastasis of tumors expressing CD73 endogenously or heterotopically, and the mice knocking out A2aR gene could be significantly protected from tumor metastasis [38]. In the present study, we found that A2aR is higher expressed in colorectal cancer rather than that in adjacent nontumor tissues (p=0.003), and the expression level of A2aR is significantly associated with tumor size (p=0.024), depth of tumor invasion (p=0.011), and TNM stage (p=0.005). Moreover, univariate analysis has shown that A2aR expression was inversely correlated with OS (p=0.017) and multivariate analysis has further confirmed that positive status of A2aR expression is an independent risk factor for the prognosis of patients (HR=2.400, 95% CI: 1.264-4.558, p=0.007).

This study showed a positive association between expressions of A2aR and PD-L1 in colorectal cancer (r=0.548, p<0.001), suggesting that A2aR and PD-L1 might have association with generation and progression of the disease. Tumor infiltrating lymphocytes (TILs) can reflect the status of a host's antitumor immune response. Blocking the PD-1/PD-L1 pathway can relieve lymphocyte inhibition and improve immune response [39]. Previous studies have shown A2aR signaling inhibits the function of multiple immune subsets, including T cells and NK cells [40]. These above studies indicate that both PD-L1 and A2aR can exert immunosuppressive effects by inhibiting the function of effector cells. Recent studies in vitro have shown that the combination of a novel A2aR antagonist (PBF-509) with anti-PD-1 or anti-PD-L1 treatment can enhance the reactivity of human tumor infiltrating lymphocytes [41]. Willingham et al. have demonstrated that combining CPI-444, an A2aR antagonist, with anti-PD-L1 or anti-CTLA-4 treatment can eliminate 90% of the tumors in mice under treatment, including restoration of immune responses in models that incompletely responded to anti-PD-L1 or anti-CTLA-4 monotherapy [42]. Based on these studies, we boldly propose that combined blockade of A2aR and PD-L1 may be a new approach to colorectal cancer treatment.

## 5. Conclusion

In summary, this study evaluated the expression of PD-L1 and A2aR in colorectal cancer and analyzed their relationship with clinicopathological features and prognosis of patients, respectively. The results showed that both PD-L1 and A2aR expression in tumor tissues were higher than those in adjacent nontumor tissues, and there was a positive correlation between the expression of these two proteins. Univariate analysis showed that the high expressions of PD-L1 and A2aR were inversely correlated with the OS, respectively, and multivariate analysis further confirmed that A2aR and PD-L1 were independent factors for the prognosis of patients. These results suggested that PD-L1 and A2aR might promote the generation and progression of colorectal cancer, and combined blockade of PD-L1 and A2aR may be a new approach to colorectal cancer treatment.

## Figures and Tables

**Table 1:** Expression of PD-L1 and A2aR in colorectal cancer tissues and adjacent nontumor tissues.

Variables	cases	Positive PD-L1 expression	Negative PD-L1 expression	*χ* ^2^ ^^	p-value	Positive A2aR expression	Negative A2aR expression	*χ* ^2^ ^^	p-value
Tumor tissues	204	84(41.2%)	120(58.8%)	16.302	<0.001	108(52.9%)	96(47.1%)	8.893	0.003
Non-tumor tissues	204	46(22.5%)	158(77.5%)			78(38.2%)	126(61.8%)		

Notes: the positive expression of PD-L1 was detected in 84 (41.2%) of 204 tumor specimens and in 46 (22.5%) of the 204 matched nontumor adjacent tissues. The expression status of PD-L1 in tumor tissues is significantly different from that in nontumor adjacent tissues (p<0.001). The positive expression of A2aR was detected in 108 (52.9%) of 204 tumor specimens and in 78 (38.2%) of the 204 matched nontumor adjacent tissues. The expression status of A2aR in tumor tissues is significantly different from that in nontumor adjacent tissues (p=0.003).

**Table 2:** Correlation between clinicopathologic factors of colorectal cancer patients with the expressions of PD-L1 and A2aR in tumor tissues (p-values were calculated by *χ*^2^ test.).

Characteristic	N=204	PD-L1(-)	PD-L1(+)	*χ* ^2^	p-value	A2aR(-)	A2aR(+)	*χ* ^2^	p-value
Age(years)				1.189	0.276			1.510	0.219
≤60	76	41	35			40	36		
>60	128	79	49			56	72		
Gender				0.320	0.572			0.928	0.335
Male	124	71	53			55	69		
Female	80	49	31			41	39		
Location				1.679	0.195			0.716	0.398
colon	132	82	50			65	67		
rectum	72	38	34			31	41		
Size (cm)				1.884	0.170			5.113	0.024
≤5.0	104	66	38			57	47		
>5.0	100	54	46			39	61		
Differentiation				2.990	0.224			1.548	0.461
Well	18	14	4			10	8		
Moderate	164	94	70			78	86		
Poor	22	12	10			8	14		
Depth of tumor invasion				2.566	0.109			6.429	0.011
T1/T2	40	28	12			26	14		
T3/T4	164	92	72			70	94		
Lymph node metastasis				7.438	0.006			1.853	0.173
No	113	76	37			58	55		
Yes	91	44	47			38	53		
Distant metastasis				1.135	0.287			0.878	0.349
No	185	111	74			89	96		
Yes	19	9	10			7	12		
TNM stage				6.062	0.014			8.069	0.005
I/II	106	71	35			60	46		
III/IV	98	49	49			36	62		

Notes: detailed clinicopathological characteristics of the patients; correlation between clinicopathologic factors of colorectal cancer patients with the expressions of PD-L1 and A2aR in tumor tissues, respectively. The expression of PD-L1 was significantly correlated with lymph node metastasis (p=0.006) and tumor TNM stage (p=0.014). A2aR expression was significantly correlated with tumor size (p=0.024), depth of tumor invasion (p=0.011), and TNM stage (p=0.005).

**Table 3:** Relationship between PD-L1 and A2aR expression in colorectal cancer (p-value was calculated by Spearman correlation test).

Variables	Positive A2aR expression	Negative A2aR expression	Total	r-value	p-value
Positive PD-L1 expression	56	28	84	0.548	<0.001
Negative PD-L1 expression	52	68	120		
Total	108	96	204		

Notes: PD-L1 expression was positive in 84 cases, of which 56 cases were also positive for A2aR (66.7%), and the remaining 28 cases were negative for A2aR (33.3%). Of the 120 PD-L1-negative expression specimens, 68 cases were also negative for A2aR (56.7%), while the remaining 52 cases were positive for A2aR (43.3%).

**Table 4:** Univariate analysis of clinicopathologic factors for overall survival (OS) in 204 colorectal cancer patients (p-value was calculated by Kaplan-Meier method and log-rank test).

Characteristic	N=204	No. of death	No. of alive	*χ* ^2^	p-value
Age(years)				1.716	0.190
≤60	76	13	63		
>60	128	32	96		
Gender				0.254	0.614
Male	124	25	99		
Female	80	20	60		
Location				0.038	0.845
colon	132	29	103		
rectum	72	16	56		
Size (cm)				1.997	0.158
≤5.0	104	19	85		
>5.0	100	26	74		
Differentiation				0.828	0.661
Well	18	5	13		
Moderate	164	37	127		
Poor	22	3	19		
Depth of tumor invasion				5.028	0.025
T1/T2	40	4	36		
T3/T4	164	41	123		
Lymph node metastasis				18.061	<0.001
No	113	13	100		
Yes	91	32	59		
Distant metastasis				140.763	<0.001
No	185	31	154		
Yes	19	14	5		
TNM stage				31.582	<0.001
I/II	106	8	98		
III/IV	98	37	61		
PD-L1				9.329	0.002
Negative	120	17	103		
Positive	84	28	56		
A2aR				5.735	0.017
Negative	96	14	82		
Positive	108	31	77		

Notes: depth of tumor invasion (p=0.025), lymph node metastasis (p<0.001), distant metastasis (p<0.001), tumor TNM stage (p<0.001), PD-L1-positive status in tumor (p=0.002), and A2aR-positive status in tumor (p=0.017) were significantly correlated to OS (p<0.05).

**Table 5:** Multivariate analysis of prognostic variables in 204 colorectal cancer patients (p-value was performed using Cox proportional hazard model).

Characteristic	N=204	*β*	Hazard ratio	95% CI	p-value
Distant metastasis		2.718	15.151	6.502-35.303	<0.001
No	185				
Yes	19				
TNM stage		1.434	4.195	1.869-9.415	0.001
I/II	106				
III/IV	98				
PD-L1		0.649	1.914	1.031-3.553	0.040
Negative	120				
Positive	84				
A2aR		0.875	2.400	1.264-4.558	0.007
Negative	96				
Positive	108				

Notes: potential prognostic factors were selected based on univariate results (p<0.05) to conduct multivariate analysis using Cox proportional hazards regression models. Positive PD-L1 status (HR=1.914, 95% CI:1.031-3.553, p=0.040), positive A2aR expression (HR=2.400, 95% CI: 1.264-4.558, p=0.007), distant metastasis (HR=15.151, 95% CI: 6.502-35.303, p<0.001), and TNM stage (HR=4.195, 95% CI: 1.869-9.415, p=0.001) could be independent prognostic predictors for colorectal cancer patients.

## Data Availability

The data used to support the findings of this study are available from the corresponding author upon request.
